# Supplementary figures and images for: Accuracy of COVID-19 relevant knowledge among youth: Number of information sources matters
Source: PLoS One. 2022 Dec 27;17(12):e0267871. doi: 10.1371/journal.pone.0267871 (PMC9794086; doi:10.1371/journal.pone.0267871)

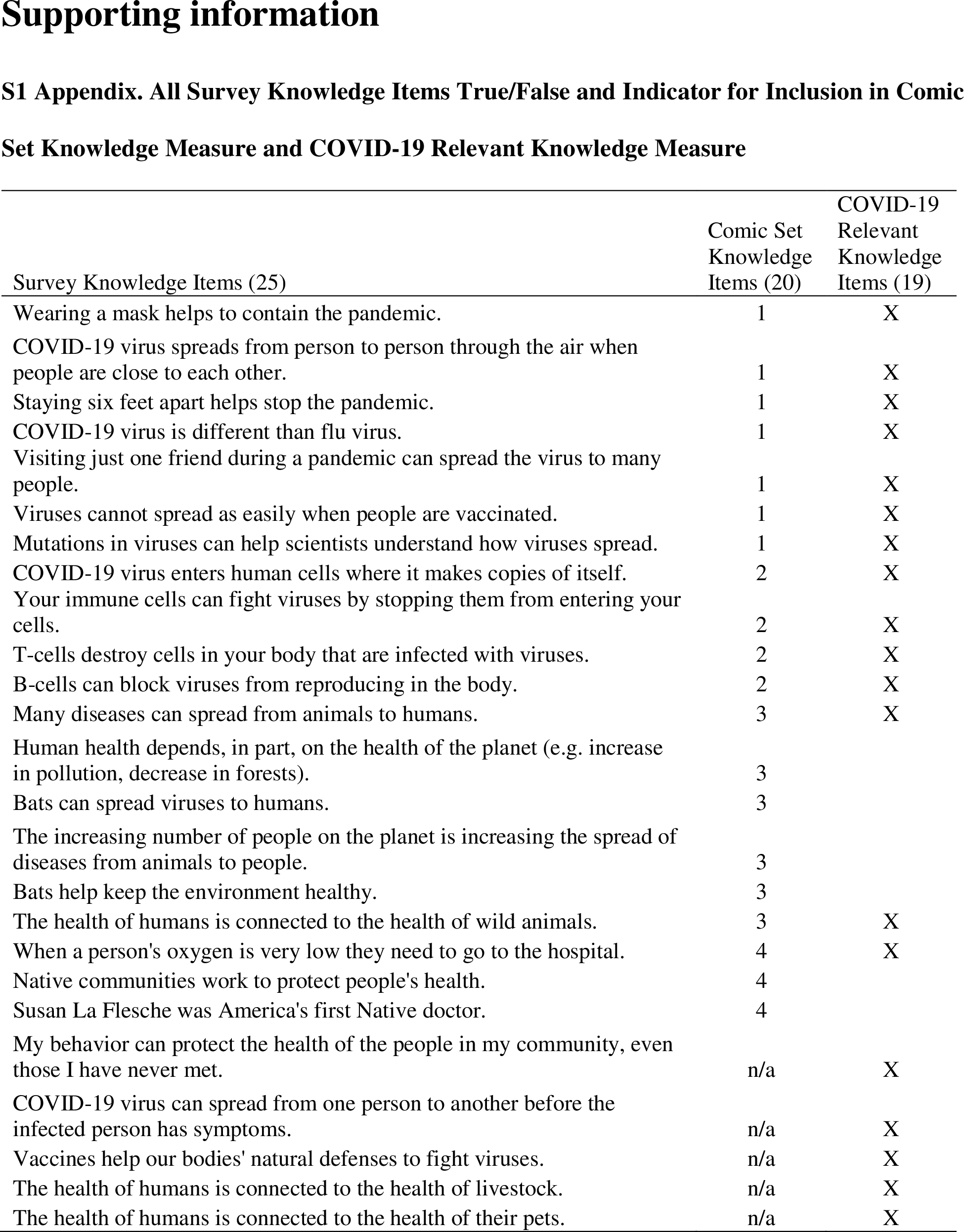

Supplement: S1 Appendix — (TIF) [file pone.0267871.s001.tif]
